# Assessing the Impact of the 2024 National Student Psychiatry Conference on Medical Student Career Aspirations and Subspecialty Interest

**DOI:** 10.1192/bjo.2024.331

**Published:** 2024-08-01

**Authors:** Dayo Taslim, Karthik Venkateswaran, Oscar Han, Abdullah Ahmad

**Affiliations:** School of Medicine and Population Health, University of Sheffield, Sheffield, United Kingdom

## Abstract

**Aims:**

The 2024 National Student Psychiatry Conference, hosted at the University of Sheffield with the theme 'Me, Myself and I,' explored the intersection of the ‘self' and the ‘other.' It delved into the dynamics of individuals in the context of their lived experiences, environment, and emerging paradigms within psychiatry and beyond. Talks and workshops aimed to heighten attendees’ interest in psychiatry by challenging societal stigma and traditional norms and expanding their perspectives of psychiatry.

**Methods:**

The pre-conference questionnaire included attendees’ year of study, university/NHS trust affiliation, current likelihood of pursuing psychiatry and career aspirations, knowledge of conference themes, and ten subspecialties represented at the conference via a faculty carousel. The post-conference questionnaire enquired about changes to the above aforementioned factors, to explore changes in career aspirations. Standardized dropdown options were used in both forms to facilitate data evaluation.

**Results:**

71 attendees were included in the final evaluation; 17 were excluded due to duplication or not completing both forms. Of the attendees, 31% were in their pre-clinical years, 56% were in their clinical years, and 4% were doctors. 9% of the participants were non-medical attendees.

Demographics of attendees included a majority from Yorkshire and Humber (52%), Midlands (11%), South England (6%), North England (10%), North East (8%) and Others (13%).

21% of attendees had been to a prior psychiatry-related conference and 34% were currently taking part in or had completed a psychiatry-related project in the past.

The level of interest in attendees aspiring to pursue psychiatry increased from 62% to 72%. An increased interest in medical psychotherapy (82%), forensic psychiatry (68%), and perinatal psychiatry (67%) after the faculty carousel was observed.

Following the conference, 97% reported increased knowledge of each theme. Findings from the faculty carousel revealed that, on average, over 90% of attendees reported an increased understanding of each speciality represented.

**Conclusion:**

The National Student Psychiatry Conference plays a significant role in increasing exposure of psychiatry to medical students and increasing their understanding of the diverse career paths within the speciality. The conference fosters networking opportunities and facilitates meaningful connections within the field, positively influencing attendees' considerations and perceptions.

